# Contour of the Face, Continued

**Published:** 1848-01

**Authors:** J. Allen


### CONTOUR OF THE FACE—CONTINUED,

BY J. ALLEN, D. D. S.

The last four named muscles, may often be sufficiently restored to their proper position, by means of artificial teeth, with gums attached, either to single teeth, or in blocks, as the case may require; but the three former, viz: the zigomaticus major, zigomaticus minor, and the massetor, cannot be brought out to any extent in this way, as the size and weight of the teeth or blocks prove objections that cannot be overcome. I have found that in a large majority of cases, it was only necessary to bring the gold appendage as far forward as the second bicuspides, thereby raising the zigomaticus major, minor, and massetor muscles, to their proper position upon the superior maxillary arch, and also the buccinnator upon the inferior arch, for at this point we meet with the swell of the anguli oris, and orbucularis oris, which are large and fleshy muscles, which in most cases require but very little raising, while the former muscles may require very much, in order to produce a symmetrical rotundity of the cheek; thus showing the beautiful harmony that may be produced, by skillfully blending the work of art with nature. So perfectly has this been accomplished within the last three years, that not even the nearest friends to those who have had it done, have been able to detect it, especially in cases where the persons having it done, have started, immediately after its completion, upon a journey, for upon their return, their improved appearance has been attributed to their very pleasant trip, change of air, &c.

The appendage by which this important change in the face is effected, is made of fine gold, and so constructed, and connected with the teeth and surrounding parts, as to bring *out* each muscle that may have fallen *in*, to its proper position, and permanently sustain it there, without inconvenience. The form of this appendage is as various as the cases are numerous, which must be shaped according to circumstances, the true indication of which is obtained by moulding the cheek upon softened wax, to whatever degree of rotundity may be desired, and casts taken therefrom, upon which the artificial structure must be made, which, for want of the necessary cuts, &c., I cannot here fully represent. It is now between two and three years since this principle was fully developed and tested. Althongh some important improvements have been made upon it, since its first introduction to the Dental profession, yet thus far it stands the test most nobly, and fully meets the fondest anticipations of its inventor.

From past experience, and present indications, I have no doubt but that from this time henceforward, this invention will be regarded as an important acquisition to the Dental profession, and here let me remark, that there are yet other important improvements, that ought, and I believe will be made, before our profession arrives to that summit of perfection, to which it is destined to reach. Let our march be onward and upward. Let us bring to our aid science, genius, and skill, together with a thorough knowledge of all that is in any way connected with our art, and let us add to these integrity, sobriety, and that gentlemanly deportment, which our profession demands, and we shall then occupy that position, which will reflect honor and profit upon ourselves, and essential benefit upon the community in which we live.

